# Projections of the insular cortex to orbitofrontal and medial prefrontal cortex: A tracing study in the rat

**DOI:** 10.3389/fnana.2023.1131167

**Published:** 2023-04-20

**Authors:** Mathias L. Mathiasen, John P. Aggleton, Menno P. Witter

**Affiliations:** ^1^School of Psychology, Cardiff University, Cardiff, Wales, United Kingdom; ^2^Kavli Institute for Systems Neuroscience, Egil and Pauline Braathen and Fred Kavli Center for Cortical Microcircuits, NTNU Norwegian University of Science and Technology, Trondheim, Norway

**Keywords:** Neuroanatomy, orbital cortex, prelimbic cortex, infralimbic cortex, dorsal peduncular cortex, anterior cingulate cortex, gustatory area

## Abstract

The dense fiber pathways that connect the insular cortex with frontal cortices are thought to provide these frontal areas with interoceptive information, crucial for their involvement in executive functions. Using anterograde neuroanatomical tracing, we mapped the detailed organization of the projections from the rat insular cortex to its targets in orbitofrontal (OFC) and medial prefrontal (mPFC) cortex. In OFC, main insular projections distribute to lateral and medial parts, avoiding ventral parts. Whereas projections from the primary gustatory cortex densely innervate dorsolateral OFC, likely corresponding to what in primates is known as the secondary gustatory cortex, these projections avoid mPFC. Instead, mPFC is targeted almost exclusively by projections from agranular fields of the insular cortex. Finally, “parietal” domains of the insular cortex project specifically to the dorsolateral OFC, and strongly innervate ventral portions of mPFC, i.e., the dorsal peduncular cortex.

## Introduction

**Figure 1:**
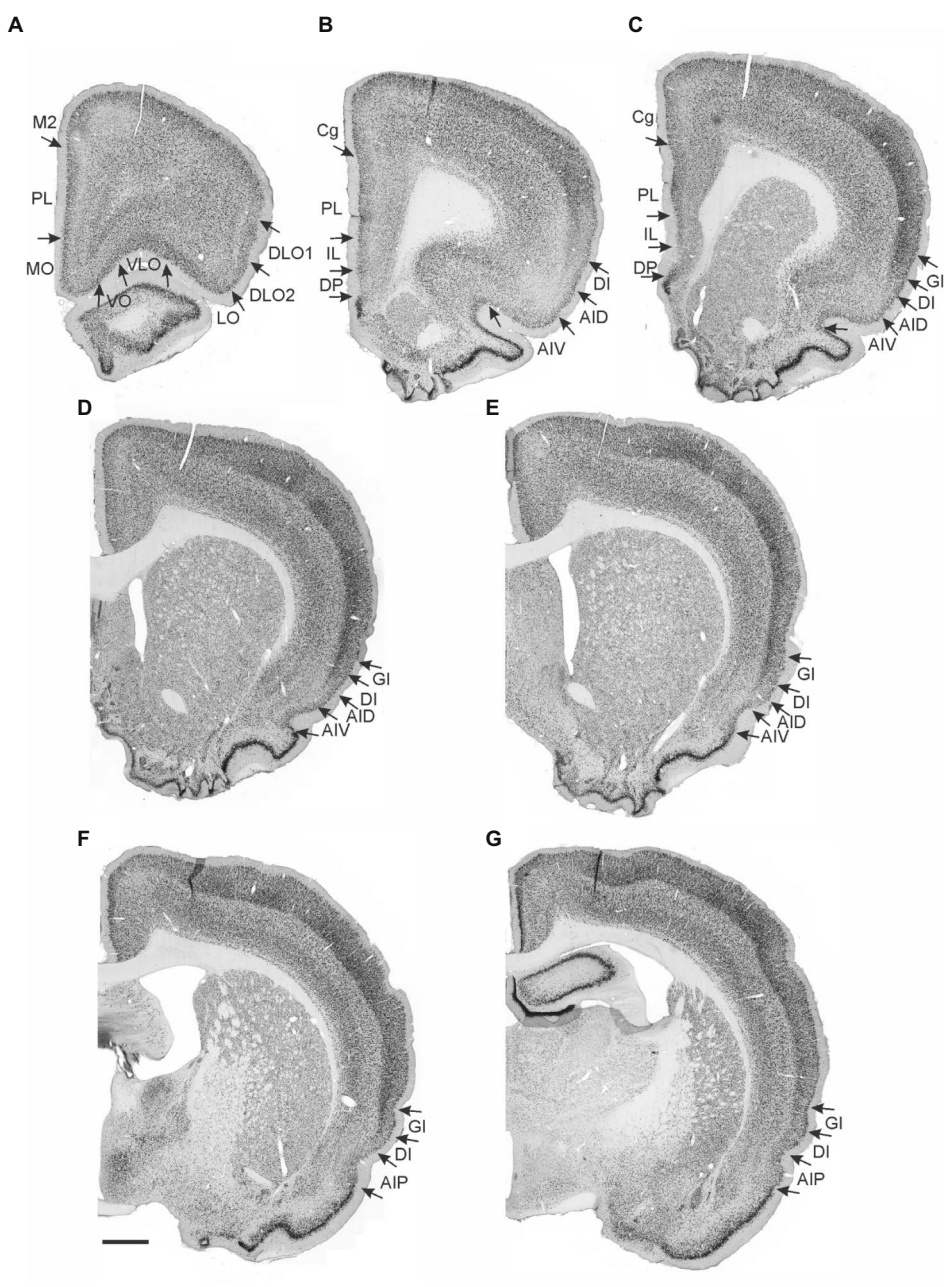
Photomicrographs of NeuN-stained coronal sections of the rat brain taken at different rostrocaudal levels through the frontal part of the brain. The coronal sections are arranged from rostral to caudal. **(A–C)** Most rostral sections showing the orbital fields and medial prefrontal regions and the replacement of orbital cortex with insular cortex. **(B,C)** Sections showing the anterior insular domain at rostral **(B)** and caudal positions **(C)**. **(D–E)** Sections showing the posterior insular domain at rostral **(D)** and caudal positions **(E)**. **(F–G)** Sections showing the parietal insular domain at rostral **(F)** and caudal **(G)** positions. Arrows indicate borders between cortical areas. Scale bar equals 1,000 μm. AIV, ventral agranular insular cortex; AID, dorsal agranular insular cortex; AIP, agranular parietal insular cortex; Cg, anterior cingulate cortex; DI, dysgranular insular cortex; DLO, dorsolateral orbitofrontal cortex; DP, dorsal peduncular cortex; GI, granular insular cortex; IL, infralimibic cortex; LO, lateral orbitofrontal cortex; MO, medial orbitofrontal cortex; M2, secondary motor cortex; PL, prelimbic cortex; VLO, ventrolateral orbitofrontal cortex; VO, ventral orbitofrontal cortex.

The rat insular cortex, which comprises the primary visceral and gustatory sensory cortices, processes information from a variety of sensory modalities, and is considered an interoceptive area that codes for affective states (Nieuwenhuys, 2012; Gogolla, 2017). In addition, visceromotor components have been described within the insular cortex (Yasui et al., 1991). Three insular domains, anterior, posterior, and parietal, have been identified based on hodological and cytoarchitectural criteria. These three *domains* subdivide the insular cortex along its rostro-caudal axis, whereas at least three insular *fields*, the granular, the dysgranular and the agranular fields, subdivide the insular cortex along its dorso-ventral axis (Krettek and Price, 1977; Cechetto and Saper, 1987; Shi and Cassell, 1998a,b; Paxinos and Watson, 2007; Van De Werd and Uylings, 2008; Figure 1).

All insular subdivisions have specific functional properties that are relevant for the function of the frontal cortices. Somatosensory and auditory information target the parietal insular cortex, an area implicated in fear conditioning (Rosen et al., 1992; Shi and Cassell, 1998a; Shi and Davis, 1999; Brunzell and Kim, 2001; Kimura et al., 2007; Rodgers et al., 2008). Processing of gustatory and visceral information in the posterior insular cortex takes place in the granular and dysgranular fields; the primary gustatory cortex is principally positioned within the dysgranular field (Kosar et al., 1986; Cechetto and Saper, 1987; Allen et al., 1991; Nakashima et al., 2000). The posterior insular domain is also involved in the learning of aversions to noxious tastes (Nerad et al., 1996; Shema et al., 2007; Schier et al., 2014) and has been implicated in aspects of reward-related behavior (Contreras et al., 2007; Li et al., 2013). Finally, the anterior insular domain, specifically the agranular field, has been implicated in reward-related behavior (DeCoteau et al., 1997; Ragozzino and Kesner, 1999) but is also an important part of a circuit involved in analgesia (Burkey et al., 1996, 1999; Jasmin et al., 2003).

The rat orbitofrontal (or “orbital) cortex is a frontal agranular cortical area, positioned ventral and lateral to the medial prefrontal cortex. It has been subdivided into five orbital fields, the medial (MO), ventral (VO), ventrolateral (VLO), lateral (LO) and the dorsolateral (DLO) fields (Krettek and Price, 1977; Groenewegen, 1988; Ray and Price, 1992; Reep et al., 1996; Van De Werd and Uylings, 2008). As we observed distinct labeling patterns along the dorsoventral axis of the latter field, we further subdivided it into the DLO1 and the DLO2 fields (Van De Werd and Uylings, 2008). The orbital cortex is a multisensory area which is pivotal for encoding of gustatory information, cue-reward associations, and behavioral flexibility (Rolls, 2000, 2005; Schoenbaum and Roesch, 2005; Schoenbaum et al., 2009). Furthermore, data indicate that the orbital fields are functionally specialized (King et al., 1989; Backonja and Miletic, 1991; Follett and Dirks, 1995; Schoenbaum et al., 2009; Hoover and Vertes, 2011).

Based on the apparent functional overlap between the orbitofrontal and insular cortices, and the functional separation between divisions present in both, one may expect that functionally defined equivalent divisions in both areas are selectively connected. Of particular interest is whether the primary gustatory cortex targets orbitofrontal cortex and, if so, which specific fields are targeted.

The medial prefrontal cortex in the rat comprises several regions situated on the medial wall of the hemisphere. Here we consider four regions that constitute the medial prefrontal cortex. From ventral to dorsal these are the dorsal peduncular (DP), infralimbic (IL), prelimbic (PL), and the anterior cingulate (Cg) cortices. In the rat, these regions are all agranular (Krettek and Price, 1977; Uylings and Van Eden, 1990; Uylings et al., 2003) and although they show strong interareal connectivity and share connections with the parahippocampal region (Jones et al., 2005; Jones and Witter, 2007), they display clear cytoarchitectural and hodological differences.

Functionally, the medial prefrontal cortex is strongly linked to working memory, cognitive flexibility, attention, and other executive functions, and several accounts have attempted to relate the different functions to the different medial prefrontal regions (Heidbreder and Groenewegen, 2003; Euston et al., 2012). The prelimbic and infralimbic cortices are pivotal for working memory processes and cognitive flexibility (Dias and Aggleton, 2000; Dalley et al., 2004). Moreover, in these areas, changes in firing rate of a substantial number of neurons correlate with reward and reward anticipation, which might be crucial for decision making and goal-directed behavior (Pratt and Mizumori, 2001; Burton et al., 2009; Euston et al., 2012). The infralimbic cortex is also considered a visceromotor center, which modulates visceral functions via its dense connections with autonomic subcortical centers (Vertes, 2006), and lesions in the area result in increased anxiety related behavior as well as increased resistance to extinction of conditioned fear (Heidbreder and Groenewegen, 2003). In contrast, the anterior cingulate cortex is, among other things, a pivotal hub in the cortical nociceptive system (Calejesan et al., 2000; Donahue et al., 2001; Johansen et al., 2001). In comparison with these three medial prefrontal areas, less is known about the function of the dorsal peduncular cortex.

A prevalent view of insular-medial prefrontal connections depicts the insular cortex as a viscerosensory area, which transmits information to the visceromotor medial prefrontal cortex. This model strongly relies on the dense connectivity between the agranular insular fields and the infralimbic cortex. However, although clear projections to both the prelimbic and infralimbic cortices are known to originate from the insular cortex, uncertainty surrounds the exact fiber distribution as well as the areas of origin in the insular cortex (Reep and Winans, 1982; Saper, 1982; Krushel and Van Der Kooy, 1988; Allen et al., 1991; Yasui et al., 1991; Van Eden et al., 1992; Conde et al., 1995; Shi and Cassell, 1998b; Gabbott et al., 2003; Jasmin et al., 2004; Hoover and Vertes, 2007; Zingg et al., 2014). In general, cortical input tends to target the medial prefrontal cortex in a dorsoventral pattern that does not fully ‘align’ with the regional borders, and we therefore asked whether insular cortex projections follow the same pattern.

Consequently, with the use of a series of anterograde tracer injections involving all insular divisions, we systematically report the distribution of insular cortex projections to the orbitofrontal and medial prefrontal cortex. Our data show that in this cortical network, insular divisions show specific connectional preferences for certain divisions in either medial prefrontal or orbitofrontal cortex, with the primary gustatory insular-orbitofrontal projection as a clear example.

## Materials and methods

### Animals

A total of 38 adult female Sprague Dawley (SD) rats (weight 180–290 g) were used in this study. The anterograde tracer injections analyzed in this study were also used in a previously published paper on the projections of the insular cortex to the parahippocampal region (Mathiasen et al., 2015; Table 1). SD rats were housed in individual cages with food and water available *ad libitum*. Surgical procedures were approved by the Animal Welfare Committee of the Norwegian University of Science and Technology and followed the European Communities Council Directive and the Norwegian Experiments on Animals Act. All surgical procedures were performed under isoflurane anesthesia. The animals were mounted in a stereotaxic frame (Kopf Instruments, CA, USA) and stereotaxic coordinates were derived from a stereotaxic brain atlas (Paxinos and Watson, 2007), adjusted according to the weight of the animals.

**Table 1:** Experimental data for all cases analyzed in the study.

Animal number and tracer used	Injection site	Layers
15876B	**Anterior AIV**	**V-VI** (III)
16310B	**Anterior AIV** (AID)	**V,VI** (II,III)
16311B	**Anterior AIV** (AID)	**III-V** (II)
14894B	**Anterior AIV** (LO)	**II-V**
13985B*	**Anterior AID**	**II-V**
14195P*	**Anterior AID (DI, post AID)**	**II-VI**
16435B	**Anterior AID** (DI)	**III** (II,V,VI)
16958B	**Anterior AID (AIV)**	**II-III** (V)
16993B	**Anterior AID (AIV)**	**III-V** (II)
14894P	**Anterior DI**	**V** (II,III,VI)
15874B	**Anterior DI** (GI)	**LII-VI**
16518B	**Anterior DI** (AID)	**III-V** (VI)
15307B	**Posterior AID**	**II-VI**
15622B	**Posterior AID**	**LIII-V** (VI)
15410B	**Posterior AID (DI)**	**LV** (LVI)
15610B	**Posterior AID (DI)**	**LV** (III)
16003B	**Posterior AID (DI)**	**II-V**
15627B	**Posterior AID**	**III-V**
13256P	**Posterior AIV**	**II-VI**
16694B	**Posterior AIV (AID)**	**II-III** (V-VI)
12903P	**Posterior DI**	**III-V**
13256B	**Posterior DI**	**V-VI**
15407B	**Posterior DI**	**III-VI**
15441B	**Posterior DI** (GI, S1)	**II-V**
16693B	**Posterior DI** (AID)	**V** (VI)
15382B	**Posterior GI**	**III-VI**
15442B	**Posterior GI/DI** (S1)	**II-III**
15873B	**Posterior GI/DI**	**V-VI**
14704B	**Parietal AIP**	**II-III** (V)
12949B*	**Parietal AIP** (DI)	**II-III** (V)
18075B*	**Parietal AIP/DI**	**II-V** (VI)
18079B	**Parietal DI**	**II-VI**
13232P*	**Parietal DI**	**V-VI**
13235P*	**Parietal DI**	**III-V**
13232B	**Parietal GI** (DI)	**V** (III,VI)
13017B	**Parietal GI**	**V-VI** (III,IV)
13235B	**Parietal GI**	**III-V**
13018P*	**Parietal GI**	**II-IV** (V)
13015P	**Parietal GI (DI)**	**III-IV** (II)
13015B	**Parietal GI** (DI)	**III-V**
13347P*	**Parietal GI** (S2)	**II-V**
13160B	**Parietal GI**	**V**

Left column: Identified animals (by number) with indications of tracer used for iontophoretic injection: Phaseolus vulgaris leucoagglutinin and Biotinylated dextran amine 10KD. Asterix behind the animal number indicates that the tracing data for the complete brain are available at EBRAINS (Mathiasen et al., 2020). Middle and right column: main insular division(s) and laminae injected. For each case regional and laminar position of the tracer deposit are specified. Regions and layers labeled in bold indicate the main injection sites, with bold text in brackets indicating a less extensive involvement of the specified region in the injection site. Regions and layers designated in brackets with normal font indicate a region or layer with only very minor involvement in the injection.

### Neuroanatomical tracers

We used the anterograde tracers 10kDA *biotinylated dextran amine* (BDA, Invitrogen, Molecular probes, 5% solution in 0.125 M phosphate buffer, pH 7.4) and *Phaseolus vulgaris Leucuagglutinin* (PHA-L, Vector laboratories, 2.5% solution in 10 mM phosphate buffer, pH 7.4). The anterograde tracers were iontophoretically injected via a glass micropipette (18–22 μm tip-diameter) into various portions of the insular cortex. In all cases, tracers were injected in the right hemisphere, independently of whether we injected one or more tracers in a single animal. For iontophoresis, we used an alternating (6 s on/off) current of 7 μA in case of PHA-L, and 6 μA in case of BDA. After a 7–14 d survival time, rats received a 1.8–2.0 mL intraperitoneal Equithesin injection. Equithesin is a mixture of chloral hydrate, magnesium sulphate, and sodiumpentobarbital (St Olavs Hospital pharmacy, Trondheim, Norway). Rats were transcardially perfused with a Ringer’s solution (0.85% NaCl, 0.025% KCl, 0,02% NaHCO3, pH 6.9), followed by paraformaldehyde (4% freshly prepared PFA in 0.125 M phosphate buffer, pH 7.4). The brains were removed from the skull, post-fixed for minimum 2 h in the same fixative, and stored overnight in a dimethyl sulfoxide solution (20% glycerol and 2% dimethyl sulfoxide).

### Histology

We sectioned 50 μm thick coronal brain sections with a freezing microtome (Thermo Scientific) and stored the sections in six (SD rats with anterograde injections) equally spaced series. In individual sections, we visualized BDA with fluorophore-tagged streptavidin (Invitrogen, molecular probes, Alexa Fluor 488 or 546) and PHA-L with primary (goat anti PHA-L, Vector laboratories) and fluorophore-tagged secondary (donkey anti-goat, Invitrogen, Molecular probes, Alexa Fluor 488 or 546, RRID: AB_142672 and AB_142628, respectively) antibodies. In case of animals in which both anterograde tracers were injected, we used fluorophores with different emission wavelengths to discriminate between them.

For immunohistochemical staining of anterograde tracers, we used the same procedure for all cases. Sections were rinsed 3 × 10 min in 0.125 M phosphate buffer (pH 7.4) followed by 3 × 10 min in TBS-Tx (0.5% Triton-X-100, 0.606% Tris(hydroxymethyl)aminomethane, 0.896% NaCl, pH 8.0). Sections were incubated with the primary antibody (1:1000, TBS-Tx) overnight at room temperature, rinsed 3 × 10 min (TBS-Tx) and incubated 1-2 h with the secondary antibody and/or streptavidine (1:200, TBS-Tx). Finally, sections were rinsed 2 × 5 min in a tris buffer (0.606% Tris(hydroxymethyl)aminomethane, pH 7.6) and mounted on Menzel-glass slides (Thermo Scientific) from a tris-gelatine solution (0.2% gelatin in tris-buffer, pH 7.6). Finally, slides with mounted sections were coverslipped with Entellan in a toluene or xylene solution (Merck Chemicals).

Series that were selected for Nissl staining were mounted on superfrost Menzel-glass slides from a tris solution and after drying, dehydrated in a series of ethanol solutions with increasing concentration (50, 70, 80, 90%, 2 × 100%). Sections were defatted in xylene for 2 min and rehydrated with the aforementioned ethanol concentrations in decreasing concentrations. After a quick wash under running water, sections were stained with cresyl violet. Subsequently, sections were washed in running water and differentiated in an ethanol-acetic acid solution (0.5% acetic acid, 70% ethanol), washed again in water and gradually dehydrated according to the aforementioned procedure. Finally, sections were cleared in xylene for a minimum of 5 min. Sections were coverslipped with Entellan (Merck Chemicals).

In selected cases, anterograde tracers were visualized with a non-fluorescent staining procedure. Sections were rinsed 3 × 10 min (TBS-Tx). For BDA visualization they were incubated with an ABC kit at room temperature (Vector laboratories) following manufacturer’s instructions. Sections with PHA-L were incubated with primary and secondary antibodies followed by a Peroxidase-Anti-Peroxidase procedure [PAP, 1:800 dilution in TBS-Tx; Sigma-Aldrich; (Ramos-Vara, 2005)]. A methanol/H_2_O_2_ solution was added before immunohistochemical staining in order to prevent non-specific staining. After the PAP/ABC protocol, sections were rinsed 3 × 10 min in TBS-Tx and 2 × 5 min in tris-buffer, and then incubated with 3,3′-diaminobenzidine (DAB; 10 mg in 15 mL tris buffer; Sigma-Aldrich). Directly before use, 12 μL of H_2_O_2_ (30%) was added to the solution which was subsequently filtrated.

The experimental dataset includes several brains with BDA injections that, instead of being cut at 50 μm sections with a freezing microscope, were cut in 100 μm or 150 μm sections using a vibratome (Leica Biosystems). In these cases, only one series of sections was stained directly after cutting, following the same protocol as described above for fluorescence staining. These cases were sectioned with a vibratome as this method allowed us to section additional thick brain slices from the same brains (used for analysis of synaptic connectivity, not included in this study).

### Image analysis

We scanned the distribution of labeled axons in representative sections with the aid of a MIRAX slide scanner and associated software (Zeiss MicroImaging). Neurolucida software (MBF Bioscience, Williston, VT 05495, USA) was used to plot injection sites onto a 3D template of the rat brain based on the stereotaxic brain atlas (Paxinos and Watson, 2007). Additionally, dark-field as well as fluorescence images were acquired with the aid of Neurolucida software coupled to a Zeiss Axio Imager M2 examiner. With the use of Photoshop (ADOBE CS6), the digital images were adjusted for contrast, brightness, and sharpness. The background was rendered black, and in case of fluorescence photomicrographs, color images were changed into gray-scale images. Bright-field digital files were turned into gray-scale images and inverted as to improve contrast. These image manipulations provided a uniform representation of all images in the paper and in some cases improved contrast. In one case (15307B), the representative illustrations were manually modified, removing specks of noise that could have been mistaken as fiber labeling, to best represent the actual results.

Injection sites are depicted either as schematic drawing or actual digital images of histological sections. For additional digital images of injection sites, the reader is referred to Mathiasen et al. (2015).

## Results

### Nomenclature and delineations

The nomenclature for the insular cortex and its various divisions used here is the same as in Mathiasen et al. (2015) (Figure 1). We divided the insular cortex predominantly based on cytoarchitectural criteria. The most striking cytoarchitectural characteristic of the insular cortex, separating it from the OFC anteriorly and from the perirhinal area 35 posteriorly, is the “tri-laminar” appearance of the insular cortex, which is clearly seen at low magnifications and also allows to delineate the dorsal insular border (see Figures 1F,G). This pattern is in strong contrast to the rostral appearance of perirhinal area 35, which displays a curved “bilaminar” pattern. Based on this simple criterion, we placed the caudal insular border, comparable to the previously described position, as coinciding with the caudal limit of the claustrum (Burwell et al., 1995). The rostral border coincides largely with the vanishing of the corpus callosum, in line with previous reports (Paxinos and Watson, 2007; Van De Werd and Uylings, 2008). Accordingly, the insular cortex borders the orbitofrontal cortex, specifically its dorsolateral field. Three rostrocaudal domains are present, the anterior, the posterior and the parietal insular domains (Shi and Cassell, 1998a,b). Agranular, dysgranular (DI), and granular (GI) fields are clearly present along the rostrocaudal extent of the insular cortex, except that the granular field only includes a small portion of the anterior insular cortex. The GI field is characterized by a clear granular layer 4, whereas in the DI field only a rudimentary layer 4 is observed. In the agranular fields, there is no indication of a layer 4. Two agranular subdivisions are present in each of the anterior and the posterior domains, the dorsal (AID) and the ventral (AIV) agranular fields. The AIV field is characterized by a less developed laminar organization than the AID field. The main features which distinguish the anterior, posterior, and parietal insular domains are the cytoarchitectural characteristics of the agranular fields. The AID and AIV fields in the posterior insular domain have distinct cell-sparse zones, not seen in the anterior insular domains. Furthermore, in the posterior agranular domains, layer 5 is more compact and it has smaller cells than in the anterior agranular domain. In the parietal insular domain, the agranular portion comprises only one division, the AIP field.

We divided the orbitofrontal cortex into five fields based on cytoarchitectural criteria, following previously published accounts (Figure 1A; Krettek and Price, 1977; Groenewegen, 1988; Ray and Price, 1992; Reep et al., 1996; Paxinos and Watson, 2007; Van De Werd and Uylings, 2008). The medial portion of the orbitofrontal cortex comprises the medial orbitofrontal (MO) and the ventral (VO) fields. The former is positioned on the medioventral wall of the hemisphere, while the latter is positioned lateral to MO. Centrally in the orbitofrontal cortex, one finds the ventrolateral field (VLO). The lateral field (LO) borders the VLO on the lateral side, where it extends more caudally than the other orbitofrontal fields. The dorsolateral field (DLO) is the most lateral subdivision of the region. We further subdivided DLO into two subfields, a dorsal DLO1 and a ventral DLO2 field, in line with a previous account (Van De Werd and Uylings, 2008). The DLO field is in some descriptions included as part of the insular cortex. Beside cytoarchitectural and hodological criteria, the insular cortex is defined as the “claustrocortex,” the cortical area encapsulating the claustrum. As the claustrum does not extend further anterior than the rostral tip of the genu of the corpus callosum (Mathur and Caprioli, 2009; Dillingham et al., 2019) the corresponding portion of cortex should be considered orbitofrontal (DLO) and not insular cortex.

In the medial prefrontal cortex, we identified four cortical regions in agreement with previously published accounts (Figures 1A–C; Leonard, 1969; Krettek and Price, 1977; Sarter and Markowitsch, 1983; Conde et al., 1990; Uylings and Van Eden, 1990; Freedman and Cassell, 1991; Conde et al., 1995; Uylings et al., 2003). All four regions are agranular but display clear cytoarchitectural variances. The most dorsally located region is the anterior cingulate cortex, which is bordered by the prelimbic cortex ventrally. The prelimbic cortex is characterized by well differentiated layers 2 and 3 as well as a layer 5 which, compared to the anterior cingulate cortex, is narrow and densely packed. Directly ventral to the prelimbic cortex, the infralimbic cortex is positioned, which is easily distinguished by a substantially weaker differentiation between all layers, a feature which is even more prominent in the most ventrally positioned region, the dorsal peduncular cortex.

In the following account, individual anterograde injections are referred to by a number (designating the rat used) followed by a letter, indicating the tracer used for the specific injection (B for BDA, P for PHA-L; Table 1). As is apparent from Table 1 most of the cases consisted of BDA injections, and we did not observe any notable differences between the two tracers. As a rule, in this paper we will use abbreviations for insular cortex subdivisions. For orbitofrontal and medial prefrontal cortices abbreviations are used only in the figures and for the DLO1 and DLO2 subfields of the dorsolateral field. In a few circumstances however, the abbreviations are included to avoid potential misunderstandings.

**Figure 2:**
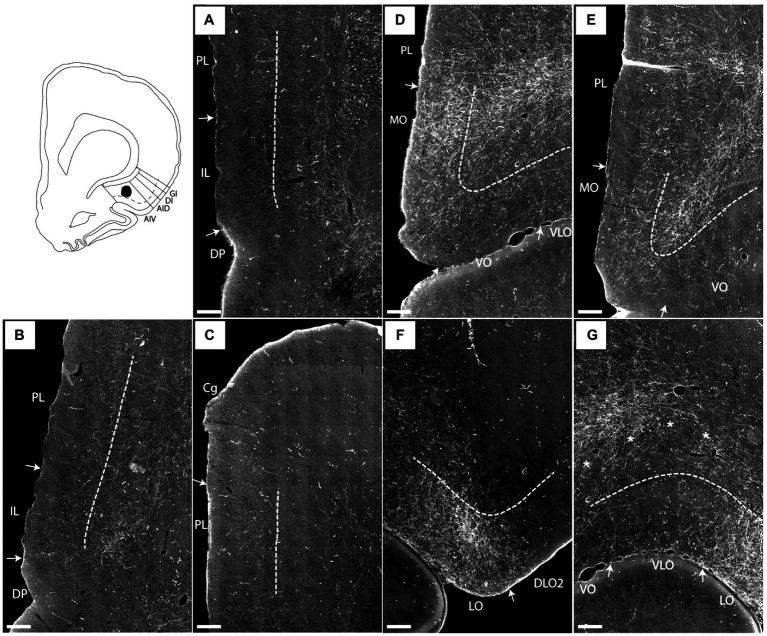
Images visualizing the fiber labeling resulting from a BDA injection covering the deep layers of the ventral agranular insular field (AIV) in the anterior insular cortex (case 15876B). A schematic depicting the injection is shown in the upper left corner. **(A–C)** Moderately dense labeling is seen in infralimbic cortex (IL), extending into the dorsal peduncular cortex (DP). More moderate diffuse labeling is seen in prelimbic cortex (PL) with a weak-to-moderate labeling in the anterior cingulate cortex (Cg). **(D,E)** At rostral portions more dense and focused fiber labeling is present in the ventral portion of the prelimbic cortex extending into an even denser labeled fiber plexus in the medial orbital field (MO). **(F,G)** Additional dense labeling is present in the lateral orbital field with only weak fiber labeling in other orbital fields. Dashed lines indicate the border between layers 3 and 5. The asterisks in G indicate the deep border of VLO and VO. VLO, ventrolateral orbital field; VO, ventral orbital field. Scale bars equal 250 μm.

We describe the varying labeling densities qualitatively as either ‘weak’, ‘moderate’ or ‘dense’. As an exemplification of the use of the intermediate term ‘moderate’, the reader is referred to the prelimbic labeling in Figures 2A–C. The rather scattered label is distributed over a large area, and although not providing a very densely packed fiber plexus (as for example the MO labeling in Figure 2D), the prelimbic labeling is far from negligible.

### Anterior AIV

Four BDA injections involved the anterior AIV (14894B, 16310B, 16311B, 15876B). One of these injections included a small portion of the anterior AID (16311B), whereas the injections in the other three cases were restricted to the anterior AIV, except for an involvement of a few single cells in lateral orbital field in one case (14894B). Because of tissue quality, in case 16310B, only OFC labeling could be analyzed.

In case 15876B, the tracer deposit was centered in the deep layers of AIV. In the medial prefrontal cortex, this injection resulted in a moderate to densely labeled plexus primarily in caudal levels of the infralimbic cortex layer 6 (Figures 2A,B). This plexus in the infralimbic cortex extended ventrally into the dorsal peduncular cortex. At this level, the moderate labeling in prelimbic cortex was relative diffuse and scattered in all cellular layers (Figures 2A,B). The rather diffuse and scattered labeling in the prelimbic cortex extended rostrally (Figure 2C), though in some sections a more densely labeled plexus was seen in the most ventral portion, continuing into an even denser labeling in the ventrally positioned medial orbitofrontal cortex (Figure 2D). This dense labeling extended to all layers but was especially dense in layer 6. Also, a weak-to-moderate labeling was seen in all layers of the rostral portion of the anterior cingulate cortex, with a preference for layer 6 (Figure 2C). The relative densely labeled plexus in the medial orbitofrontal cortex involved all layers of the area (Figures 2D,E). Furthermore, moderate to dense labeling was present in the lateral orbitofrontal cortex, primarily in the superficial layers. The labeled terminal plexus was densest in layer 3, with weaker labeling in layer 1 and even weaker labeling in layer 2 (Figure 2F). At caudal levels the deeper layers were also labeled. In the remaining orbitofrontal fields, in general only weak labeling was seen in the superficial layers of the ventral and ventrolateral orbital fields (Figures 2E,G), although at most rostral levels moderate labeling was present in layers 1 and 3 of the ventral field. The dorsolateral field only contained very few labeled fibers in the ventral portion, in subfield DLO2, close to the lateral orbital border (Figure 2F).

**Figure 3:**
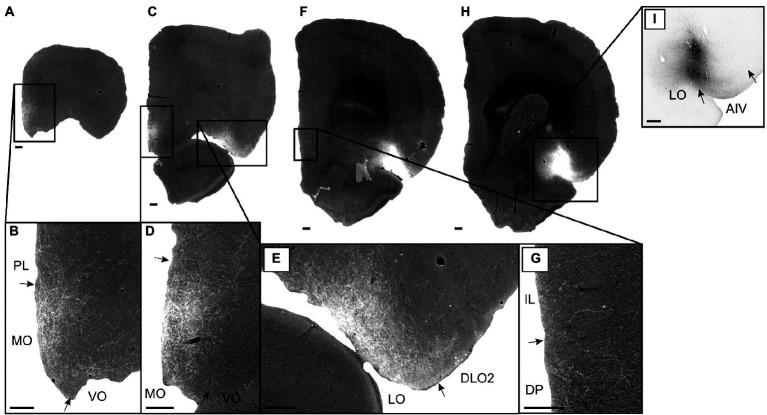
Images visualizing the fiber labeling resulting from a BDA injection covering layers 2–5 of the ventral agranular insular field (AIV) in the anterior insular domain (case 14894B). The injection also involved a small portion of the lateral orbital field (LO). **(A,B)** Low magnification **(A)** and high-magnification **(B)** images visualizing the dense fiber labeling in the medial orbital field (MO) at very rostral levels. **(C–E)** Low magnification **(C)** and high-magnification **(D,E)** images visualizing the dense fiber distribution in the medial orbital **(D)** as well as lateral orbital **(E)** fields at slightly more caudal levels. **(F,G)** Low magnification **(F)** and high-magnification **(G)** photomicrographs visualizing fiber distribution in the infralimbic (IL) and dorsal peduncular (DP) cortices. **(H,I)** low-magnification **(H)** and high-magnification **(I)** images of the BDA injection site in AIV, showing some LO involvement. Black boxes indicate the areas with zoom images. DLO, dorsolateral orbital field; PL, prelimbic cortex; VO, ventral orbital field. Scale bars equal 250 μm.

In case 14894B (Figure 3), the tracer deposit was positioned in layers 2–5 of AIV (with weak involvement of the lateral orbital field; Figures 3H,I). This injection resulted in a densely labeled plexus in the medial orbital field, with labeling in all layers, but with the densest plexus in layers 1–3 (Figures 3A–D). Furthermore, moderate to dense labeling was present in the lateral orbital field, centered in the superficial layers, especially layer 1 (Figures 3C,E). In the remaining orbitofrontal areas, very weak labeling was seen in the most ventral portion of the dorsolateral field, in the superficial layers of the DLO2, close to the border to the lateral orbital field, and in layer 1 of both the ventral and ventrolateral field at the most rostral levels. In the mPFC, we observed a weak-to-moderately labeled plexus in the infralimbic and dorsal peduncular cortices (Figures 3F,G). The labeling was primarily located in the superficial layers, especially layer 1. This contrasted with the denser fiber plexus seen in case 15876B, where both injection and fiber plexus were concentrated in the deep layers. Substantially weaker and scattered labeling was seen in the dorsal portion of the prelimbic cortex, also primarily in the superficial layers. Only few scattered fibers were present in the anterior cingulate cortex.

A very similar pattern of mPFC labeling was seen in case 16310B which received an injection centered in the deep layers of anterior AIV, involving a very small portion of the AID (for this case, only mPFC labeling was analyzed due to tissue damage in OFC). A similar labeling pattern in mPFC was observed following an injection centered in layers 3–5 (with a weaker involvement of layer 2) of AIV (16311B), also including a small portion of the deep layers of AID (not illustrated). However, in this latter case, we observed additional dense labeling in the prelimbic cortex.

It is thus apparent that anterior AIV strongly projected to the infralimbic and adjacent dorsal peduncular cortex whereas projections to prelimbic cortex were comparably weaker, except for most ventral levels of the region. Anterior AIV did seem to project to anterior cingulate cortex as well. These projections targeted all layers, although subtle differences in laminar preferences in the various medial prefrontal regions were apparent. Projections to the orbitofrontal cortex mainly reached superficial layers of the medial and lateral fields. Regarding the origin of these projections in AIV, our injections included all cellular layer of AIV and thus lack the resolution to formulate a conclusion, but it seemed likely that neurons in layers III and V were important, though not exclusive contributors to this projection.

### Anterior AID

Five injections were positioned in the anterior AID. Two injections were centered in the area with either no (13985B) or extremely limited (16435B) involvement of other insular divisions, whereas the other three cases included small portions of either the anterior DI and posterior AID (14195P) or the anterior AIV (16958B; 16993B).

**Figure 4:**
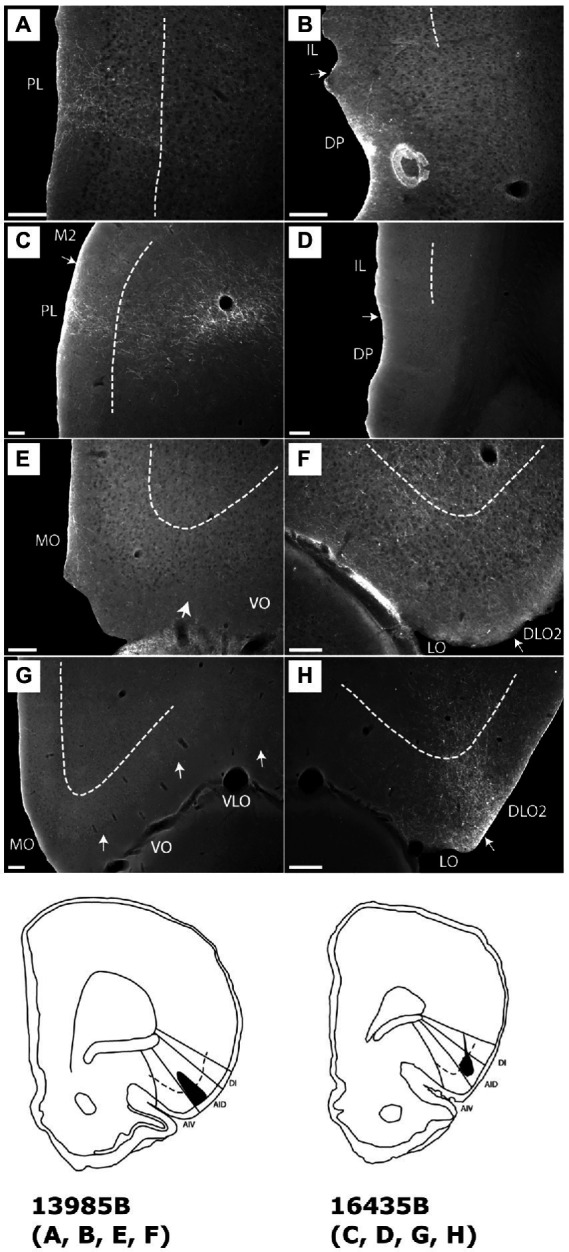
Images showing the fiber distribution in medial prefrontal and orbitofrontal cortex following BDA injections into the dorsal agranular insular field (AID) in the anterior insular domain. Two cases with the injection sites are shown in schematics below the images. **(A)** A dense, but rather confined, fiber plexus is present in superficial layers of the dorsal prelimbic cortex (PL), specifically rostrally, whereas no, or only very limited, fiber labeling is present in other medial prefrontal regions, except for weak labeling in the dorsal peduncular cortex (DP) (case 13985B). **(B)** In the same case, only very weak labeling is seen in the dorsal peduncular cortex. **(C,D)** In another case (case 16435B) a similar pattern, as in case 13985B, is seen; dense labeling is present in a rostral portion of the dorsal prelimbic cortex **(C)** with no, or extremely weak, label in other portions of the medial prefrontal cortex **(D)**. **(E,F)** In the orbitofrontal cortex, weak labeling is present in the medial orbital field (MO) **(E)** with denser labeling in the lateral (LO) and dorsolateral (DLO) orbital fields (case 13985B). **(G,H)** In case 16435B, dense orbitofrontal labeling is restricted to the lateral and dorsolateral fields. Dashed lines indicate the border between layers 3 and 5. IL, infralimbic cortex; M2, secondary motor cortex; VLO, ventrolateral orbital field; VO, ventral orbital field. Scale bars equal 200 μm.

In a representative case (13985B; Figure 4), the injection was restricted to the anterior AID including all cellular layers except layer 6. This injection resulted in moderate-to-dense labeling in the superficial layers of the dorsal and rostral portions of the prelimbic cortex (Figure 4A). In these portions of the prelimbic cortex, labeling was primarily situated in a rather confined plexus in superficial layers with only scattered labeling in the deep layers. Almost no labeling was present in the infralimbic or the anterior cingulate cortices, while in the dorsal peduncular cortex only very weak labeling was seen in layer 1 (Figure 4B). In the other case, with an injection site also centered in the anterior AID but having limited involvement of the deep layers of anterior DI (16435B), the labeling pattern described for the representative case was essentially confirmed. Dense fiber labeling was present rostrally in the dorsal prelimbic cortex, with the same laminar and topographic pattern as in case 13985B, but with some additional labeling in the deep layers (Figure 4C). Labeling in other medial prefrontal regions was sparse or absent (Figure 4D).

In the orbitofrontal cortex, we likewise observed similar labeling in these two cases. In case 13985B, fiber labeling in the medial orbital field was in general very weak (Figure 4E) except for the most rostral portion where a moderately labeled plexus was present, primarily in layer 1 with weaker labeling in layer 2. We observed moderate labeling caudally in DLO, specifically in layer 1 of DLO2 (Figure 4F). Layers 2 and 5 showed moderate labeling with slightly weaker labeling in layer 3 and even weaker fiber labeling in layer 6. In the lateral orbital field, a labeled plexus was seen primarily in the superficial layers, centered in layer 1, with somewhat weaker labeling in layers 2–3. In the deep layer 5 of the lateral field, labeling varied from very weak to, occasionally, relative dense (Figure 4F). In the remaining orbitofrontal cortex only extremely weak and scattered labeling was present in a few sections. In case 16435B, the labeling pattern was similar with a strikingly strong labeling in the around the border between LO and DLO, although with more equally distributed label in all superficial DLO layers, along with a moderate layer 5 plexus in LO (Figures 4G,H).

Summarizing these two cases (13985B, 16435B), both displayed fiber labeling primarily in the prelimbic cortex with only extremely sparse labeling in the infralimbic, anterior cingulate or dorsal peduncular cortices. Furthermore, in both cases, dense terminal fiber labeling was present in the lateral and dorsolateral orbital fields, with sparser labeling in the medial (MO) field.

In the three remaining cases, the injection sites included small portions of other areas too, and these cases differed from the two former cases in showing labeling in the infralimbic and dorsal peduncular cortices, while showing comparable labeling patterns in the prelimbic cortex and absence of labeling in anterior cingulate cortex. In the orbitofrontal cortex, labeling was comparable to that described for the two previous cases, but with some additional labeling in the medial (MO) field (in cases 16985B and 16993B that had an injection site with some involvement of anterior AIV).

These anterograde tracing data thus showed that projections from anterior AID reached the medial prefrontal cortex, showing a clear preference for superficial layers of the rostro-dorsal portion of prelimbic cortex. Projections to the orbitofrontal cortex mainly but not exclusively innervated superficial layers of the lateral and dorsolateral subfields. The distributions of the injected tracers do not allow to reach a conclusion regarding the laminar origin of the projections.

### Anterior DI

In three cases, the injections were positioned in the anterior DI, either restricted to the area (14894P), with a very small involvement of the anterior AID (16518B), or with a few single cells also in the deep layers of the anterior GI (15874B). Combined, the injections cover all layers of the anterior DI. In none of the three cases did we observe labeling in any of the medial prefrontal regions. In contrast, in all three cases labeling was present in the orbitofrontal cortex.

**Figure 5:**
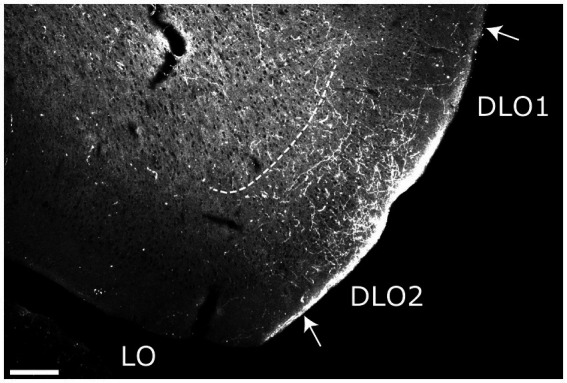
Image of a rostral section, visualizing the fiber projections to the dorsolateral orbital field (DLO) following a BDA injection in the dysgranular field (DI) of the anterior insular domain (case 16518B). Dense terminal labeling is present predominantly in superficial layers of the DLO2 field. Dashed lines indicate the border between layers 3 and 5. LO, lateral orbital field. Scale bars equal 200 μm.

Following the case with a restricted injection centered in layer 5 (14894P), only weak and scattered labeling was seen in the DLO2 field in a single section. In the other two cases (15874B, 16518B), the injection sites were in either all layers or centered in layers 3–5. In both cases, a very densely labeled plexus was present in all layers of DLO2, though at rostral levels this plexus showed a preference for superficial layers. In one of the cases (16518B; Figure 5), we noticed additional moderate DLO1 labeling as well. In both cases, fibers were also labeled in LO, primarily in layer 1, although the labeling was substantially weaker than in DLO. We did not observe labeling in any of the other orbitofrontal fields in either of these cases. As the involvement of adjacent insular portions in these two cases was very limited, and no fiber labeling was observed in medial prefrontal regions (as would be expected from anterior AID involvement), we find it most likely that the DLO fiber plexus originated from the anterior DI.

In summary, anterior DI only projected to orbitofrontal cortex, not to the medial prefrontal cortex. The targeted orbitofrontal fields were mainly DLO2 with some minor projections to LO and DLO1. The projections terminated preferentially in superficial layers and seemed to originate mainly from neurons in superficial layers of anterior DI.

### Posterior AIV and AID

Eight injections were positioned in the posterior agranular insular cortices. Two of these injections primarily involved the posterior AIV, one case with the tracer deposit restricted to the field (13256P), and one case with a considerable involvement of the posterior AID field (16694B). Six injections primarily involved the posterior AID. In three of these cases, the injection site was restricted to the posterior AID (15307B, 15622B, 15627B), whereas in the other three cases the injection sites included, in varying degrees, portions of the posterior DI (15410B, 15610B, 16003B). For two injections (15410B, 15622B), tissue quality excluded analysis of fiber distribution in the medial prefrontal cortex.

For all the cases that resulted in dense fiber labeling, the overall labeling pattern that resulted from these injections did not differ substantially, irrespective of whether the injections were centered in the dorsal or ventral agranular field. In the medial prefrontal cortex, labeling was preferentially, though not exclusively, seen in ventral prelimbic cortex, whereas labeling in anterior cingulate cortex was generally absent. In the orbitofrontal cortex the terminal fiber labeling was predominantly present in the lateral and medial orbital fields.

**Figure 6:**
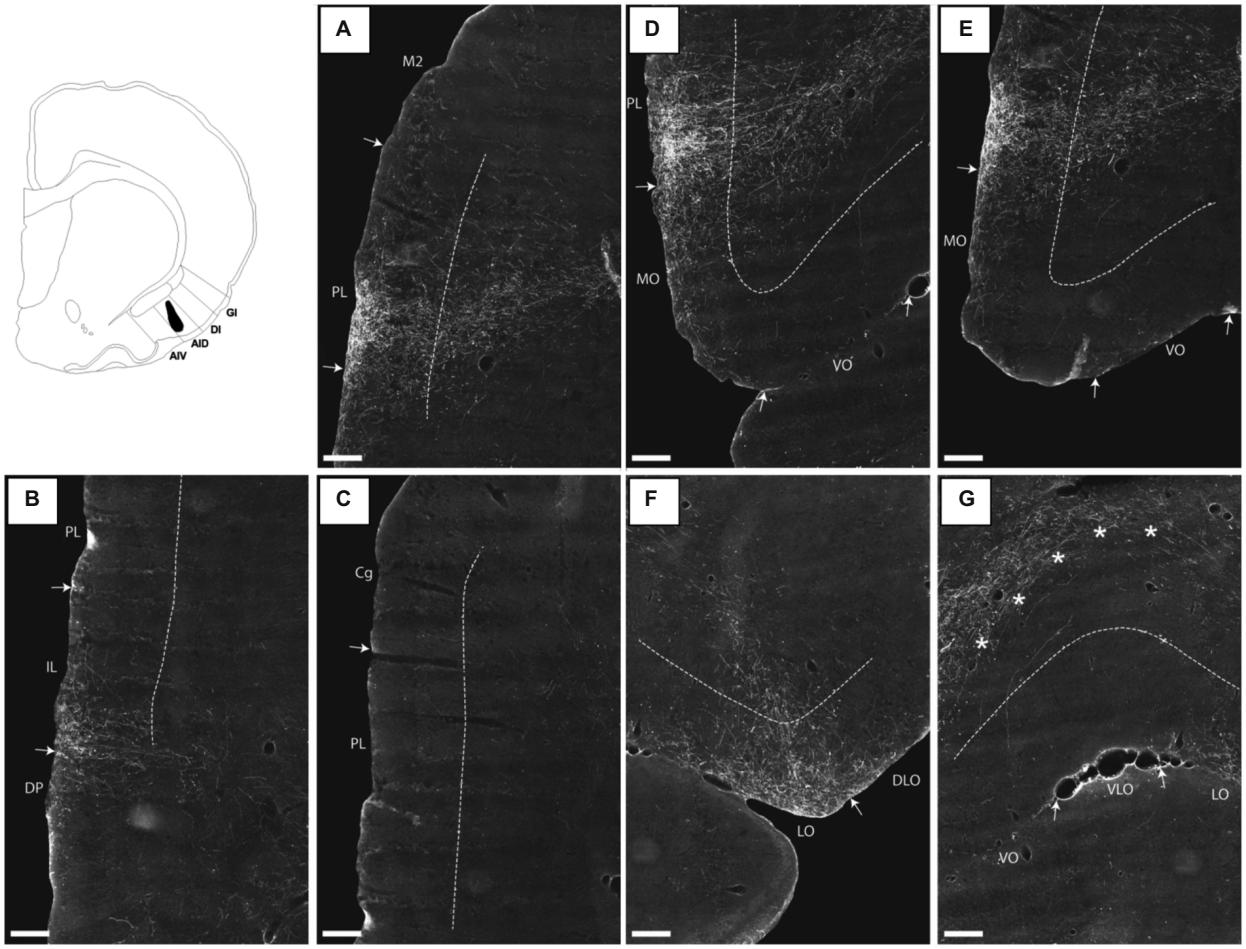
Images visualizing the fiber projections from a BDA injection covering all layers of the dorsal agranular insular field (AID) in the posterior insular domain (case 15307B). A schematic depicting the injection is shown in the upper left corner. **(A)** A dense fiber plexus is centered in the ventral portion of the prelimbic cortex (PL) at rostral levels. **(B,C)** At more caudal levels, the dense labeling targets specifically infralimbic cortex (IL) with sparser label in the dorsal peduncular cortex (DP). The image in **(C)** shows the same section as **(B)** at more dorsal levels, illustrating the diminishing of fiber labeling in prelimbic and anterior cingulate (Cg) cortices at this a-p level. **(D,E)** In medial portions of the orbitofrontal cortex, moderate fiber labeling is present in superficial layers of the medial orbital field (MO) but only very few labeled fibers are present in the ventral orbital field (VO). **(F)** A concentration of fiber labeling is present in superficial layers of the lateral orbital field (LO), with much sparser label in the dorsolateral orbital field (DLO). **(G)** Virtually no fibers are present in the ventrolateral orbital field (VLO). The dense-to-moderate labeled fiber path seen in the upper part of the image is positioned deep to layer 6 and does not target the orbitofrontal cortex. These fibers likely target the deep layers of mPFC as can also be seen in **(D)**. Dashed lines indicate the border between layers 3 and 5. The asterisks in G indicate the deep border of VLO and VO. AIV, ventral agranular insular field; DI, dysgranular insular field; GI, granular insular field. Scale bars equal 250 μm.

**Figure 7:**
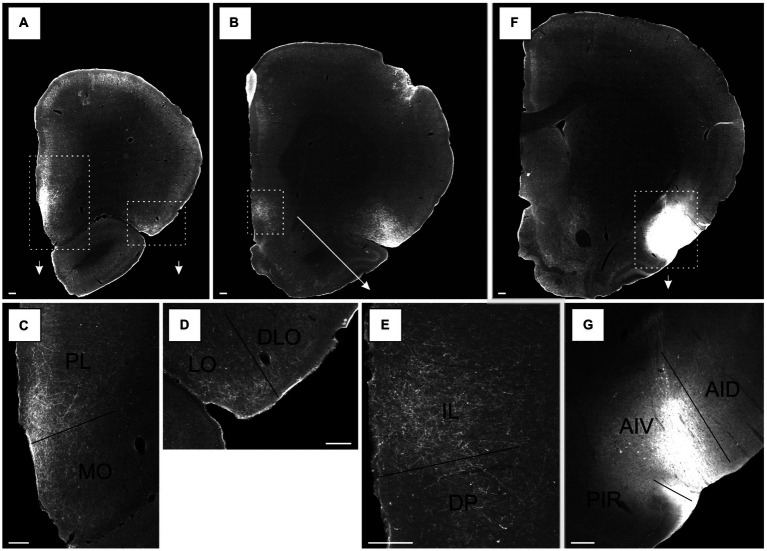
Images of the labeled projections resulting from a PHA-L injection covering all layers of the ventral agranular insular field (AIV) in the posterior insular cortex (case 13256P). **(A,B)** Low magnification photomicrographs of sections with fiber labeling in medial prefrontal and orbitofrontal cortices. Stippled white boxes indicate the areas with higher magnification images in **C–E**, associated arrows points towards the associated zoom photomicrographs. **(C)** Labeled fibers target superficial layers in a continuous band covering both the prelimbic cortex (PL) and medial orbital field (MO). **(D)** Moderate fiber labeling is distributed in the superficial layers of the lateral orbital field (LO) with substantially fewer fibers in the dorsolateral orbital field (DLO). **(E)** Dense labeling in the infralimbic cortex (IL), predominantly in the superficial layers, with substantial less fiber labeling in the dorsal peduncular cortex (DP). **(F)** Low magnification image of the position of the PHA-L tracer deposit in the AIV field. White stippled box and the arrow indicate the portion of the section highlighted in a higher resolution image in G. **(G)** The center of the injection site. Additional abbreviations: AID, dorsal agranular insular field; PIR, piriform cortex. Scale bars equal 200 μm.

In a representative case with an injection restricted to the posterior AID, including all layers (15307B), labeling was present in both the pre- and infralimbic cortices. The plexus in the prelimbic cortex was very dense, specifically in the ventral portion at rostral levels of the region. Labeling was very dense in layer 1, with more moderate labeling in all other layers (Figure 6A, see Figure 6C for comparison with more caudal levels). Similarly positioned moderate-to-dense labeling was seen in the infralimbic cortex. This plexus, although weaker, also extended into portions of the dorsal peduncular cortex (Figure 6B). Case 13256P had an injection that was restricted to posterior AIV, covering all layers of the field (Figure 7). In this case, a densely labeled plexus was present in the prelimbic cortex, restricted to the ventral portion at mid-rostral levels (Figures 7A,C). The plexus was centered in layers 1–2, with substantially weaker labeling in layer 3. In the infralimbic cortex, dense labeling was present in superficial layers with some weak labeling in deep layers (Figures 7B,E). This very dense labeling stopped at the ventral border of the infralimbic cortex, and ventral to this, labeling was substantially more moderate, primarily present in the superficial layer of the dorsal peduncular cortex. This pattern of labeling was largely replicated in a case where the injection site covered primarily layers 2–3 of both the posterior AID and AIV fields (16694B), although the labeling in the ventral prelimbic cortex showed a clear preferential distribution in layer 1. The three remaining cases resulted in no (15627B) or only weak (15610B; 16003B) labeling in mPFC (see Table 1).

Turning to the orbital fields, in the case where the injection site was restricted to the posterior AID, and involved all cell layers (15307B), a moderate plexus was present in the medial and lateral orbital fields. In both fields, the plexus was centered in layer 1 with weaker labeling of the other superficial layers, and with some involvement of layer 5, caudally in the lateral field (Figures 6D–F). In the same figures only very weak labeling can be seen in the dorsolateral orbital field, except for the very caudal levels, where a slightly more moderate innervation of the DLO2 field was present, centered in layer 1 (Figure 6F). In the ventral and ventral lateral fields, only single scattered fibers were seen, except at the very rostral level where we noticed extremely weak and scattered labeling in all layers of the ventral field (Figures 6E,G); Note that the dense fiber labeling in G is located deep to the orbitofrontal cortex; asterisks indicate the deep border of layer 6; these fibers are actually in mPFC, not in OFC (see also Figure 6D where these fibres travel to). In case the injection site included parts of both the posterior AID and AIV (16694B), and was centered in the superficial layers, a comparable labeling pattern was seen, except that the labeling was overall weaker than in the previous case and was restricted to layer 1.

An injection restricted to the posterior AIV, involving layers 2–6, the same orbitofrontal fields were labeled (13256P). In this case, a dense plexus was seen in the medial orbital field (MO) that was continuous with the plexus in the prelimbic cortex (Figures 7A,C; see Figures 7F,G, for injection site). In the medial field, labeling was seen in all the superficial layers, though being densest in layers 1–2, with substantially weaker labeling in deep layers. The labeling did not extend to the most rostral levels. In the lateral orbital field, a moderate plexus was seen in the superficial layers, centered in layer 1 with weaker labeling in layers 2–3 (Figures 7A,D). The plexus was only seen at the lateral portion of the region at the border to the dorsolateral field, and it continued into layers 1–2 of a small part of the most ventral portion of the DLO2 field, although with only very weak labeling in the latter field.

The remaining injections centered in posterior AID (see Table 1 for details) resulted in either no or weak labeling in lateral OFC, with variable involvement of LO and or DLO. In none of these cases did we observe labeling in other fields of OFC.

The anterograde tracing data lead us to conclude that posterior AIV and AID shared overall projections to superficial layers of the ventral prelimbic cortex and adjacent infralimbic cortex, with much weaker to absent projections to any of the other medial prefrontal cortical fields. Projections to the orbitofrontal cortex preferentially innervated superficial layers of the lateral and medial orbital fields.

The three cases with very dense fiber labeling all had injections that, to varying degrees, included all cellular layers. In the cases with less or no labeling, the injections involved mainly layers 3–5, thus suggesting that layer 2 neurons in AIV and AID are a prominent origin of the projections to the frontal cortices.

### Posterior DI

Five injections, which, when combined, covered the entire rostrocaudal axis and all layers of posterior DI (12903P, 13256B, 15407B, 16693B, 15441B) were available for analysis. Three of these injections were restricted to the field (12903P, 13256B, 15407B). In the two other cases, the injection site included either a small portion of posterior AID (16693B) or a small portion of the dorsally positioned posterior GI, with minor involvement of the somatosensory cortex (15441B).

We did not observe labeling in medial prefrontal cortex, beyond weak or scattered labeling, in any of the three cases with an injection restricted to the region (12903P, 13256B, 15407B). Only very weak or scattered labeling was seen in the ventral portion of the prelimbic cortex with even more sparser labeling in the dorsal portion of the infralimbic cortex. In none of these cases did we observe labeling in the anterior cingulate cortex. A similar labeling pattern was seen when the injection site included a small portion of the posterior GI field (15441B). Finally, when the injection site included a small portion of the posterior AID (16693B), besides the weak labeling in ventral prelimbic and dorsal infralimbic cortices, a moderate plexus was present in the prelimbic cortex rostrally, comparable to the pattern described for posterior AID injections.

**Figure 8:**
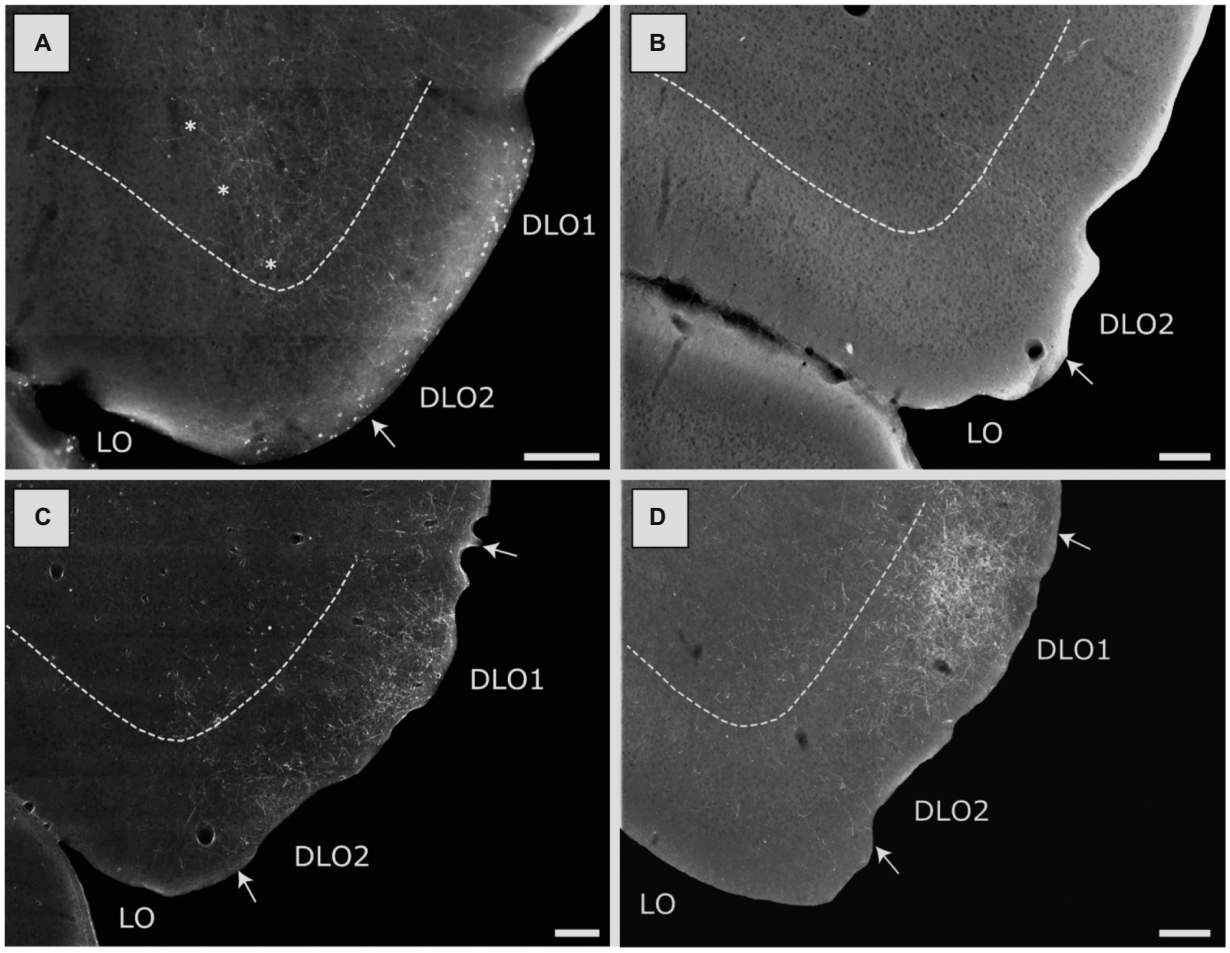
Image of labeled fibers in the lateral (LO) and dorsolateral (DLO) orbital fields, resulting from anterograde tracer injections into various portions of the insular cortex. **(A)** Distribution of terminal labeling following a BDA injection in the DI field of the posterior insular domain (case 15407B). Labeled fibers are particularly dense in the DLO2 field with more moderate labeling in the LO field. Asterisks indicate the LO/DLO border. **(B)** Distribution of terminal labeling following a BDA injection in the GI field of the posterior insular domain (15442B). Only very sparse labeling can be seen, located to the DLO2 field. **(C)** Distribution of terminal labeling following a BDA injection in the DI field of the parietal insular domain (18079B). In this case labeling is concentrated in both the DLO1 and DLO2 fields with virtual no labeling in other orbital fields. **(D)** Distribution of terminal labeling following a PHA-L injection in the GI field of the parietal insular domain (13347P). Although fiber labeling covers both DLO fields, it is heavily concentrated in DLO1. Among other orbital fields, only LO is sparsely labeled. Dashed lines indicate the border between layers 3 and 5. Scale bars equal 200 μm.

In contrast, dense labeling was present in the orbitofrontal cortex, particularly the dorsolateral field, and as was the case for anterior DI injections, the laminar position of the injections apparently determined whether we observed this labeling. In two cases, the injections involved either layers 2–5 (15441B) or layers 3–6 (15407B). In both, we observed dense or moderate labeling in the dorsolateral field, particularly at caudal levels of DLO2 (Figure 8A). These injections also resulted in moderate labeling in layers 1–5 of caudo-lateral portions of the lateral orbital field with a preference for superficial layers. Virtually no fibers were present in the medial, ventral or ventral lateral fields in these two cases. In other cases, we did not observe dense labeling. Following an injection restricted to the deep layers (13256B), we did not observe labeling in the orbitofrontal cortex, and an injection in layers 3–5 (12903P) displayed weak labeling in the DLO2 field with even weaker and scattered labeling in the lateral field. An animal with an injection in the deep layers of posterior DI, which also included a small portion of the posterior AID (16693B), had only scattered labeling in the lateral and dorsolateral fields, except for a weakly labeled plexus in the dorsal DLO2, at most rostral levels. In line with the very small involvement of posterior AID of the injection, scattered fibers were also labeled in the medial orbital field.

Our tracing data thus indicate that posterior DI did not project to medial prefrontal areas. Projections to orbitofrontal cortex originated mainly from superficial layers. These projections targeted mainly superficial layers of fields at more caudal levels, including LO and DLO2. In the latter field, projections also innervated deeper layers. The overall distribution of projections from posterior DI were thus quite similar to those described above for the anterior DI field, although the anterior portion also projected to field DLO1.

### Posterior GI

Three injections were centered in the posterior GI, with one of the injections being restricted to GI (15382B) and the two other injections including a portion of the posterior DI (15442B, 15873B). The three injections combined included all cell layers. Fiber labeling was largely absent in the medial prefrontal cortex in any of these cases. Regarding labeling in the orbitofrontal cortex, in only one case (15442B), with an injection in layers 2–3, including a substantial portion of DI, we observed very weak labeling in the dorsolateral field (DLO2 subfield), at most caudal levels (Figure 8B). In the two remaining cases the tracer deposits covered layers 3-6and resulted in no labeling in the orbitofrontal cortex.

We conclude that the posterior GI did not contribute to insular projections to orbitofrontal and medial prefrontal cortical areas.

### Parietal DI

In three cases the tracer deposits were centered in and restricted to the parietal DI. The injections covered either all layers (18079B), layers 3–5 (13235P), or layers 5–6 (13232P). Contrary to what was observed in cases with AIP injections, we did not observe dense labeling in the dorsal peduncular cortex following injections in parietal DI. In the single case where the injection site covered all layers, only a very weak innervation of the superficial layers, primarily layer 1 of the dorsal peduncular cortex was present. In this case virtually no fibers were seen in other regions of the medial prefrontal cortex, except for very few single scattered fibers in the infra- and prelimbic cortices. In the other two cases, the overall distribution of the labeling was similar though even weaker. In none of the cases did we observe labeling in the anterior cingulate cortex.

In the orbitofrontal cortex, labeling was seen in the superficial layers of the dorsolateral field (both DLO1 and DLO2) in all three cases. In the two cases with injections in either all layers or layers 3–5, the labeled plexus was centered in layer 1 or layers 1–2, with a substantially weaker involvement of layers 3–5 (Figure 8C). In these two cases, no labeling was observed in any other of the orbitofrontal fields except few scattered fibers in the lateral orbital field. In the case with a deep layer injection (13232P), numerous labeled scattered fibers covered all superficial layers of the dorsolateral field. Labeled fibers were also observed in other orbitofrontal fields, specifically the lateral and medial fields. This latter labeling was only observed in a few sections and was, especially for the medial fields, quite weak.

Our results thus indicate that parietal DI had only very minor projections to medial prefrontal areas and the projections to orbitofrontal fields are almost limited to DLO1 and DLO2, with a preferred termination in layers 1 and 2. Our anterograde data do not allow to conclude which layers originate the projections to the dorsolateral orbitofrontal cortex.

### Parietal GI

Eight injections were centered in the parietal GI. In two cases (13347P, 13018P), the injection sites included, in varying degrees, all layers except layer 6, and in these cases a very densely labeled plexus was present in the dorsolateral orbital field, primarily in superficial layers (Figure 8D, case 13347P). The plexus included both DLO1 and DLO2 though with the densest labeling in dorsal DLO1. In case 13018P, only weak and scattered labeling was observed in layer 3 of the dorsolateral field with additional weak labeling in layer 5. Weak labeling was observed in layers 1–2 of the lateral orbital field. Labeling in the lateral field showed a slight increase in density at very caudal levels. No other orbitofrontal fields were labeled except for a few fibers in the ventrolateral field (case 13018P).

Three other cases with the injection sites either positioned solely in layer 5 (13160B), with a further minor involvement of all other layers except layer 2 (13232B) or restricted to layers 3–5 (13235B) did not result in labeling in any of the orbitofrontal fields. In the remaining three cases (13015P; 13015B; 13017B), the injection sites covered all layers except for layer 2, with one exception where a few cells were labeled in layer 2 (13015P). In two of these cases, the tracer deposit involved either a substantial (13015P) or a minor (13015B) portion of the parietal DI. Irrespective of the slight variation in injection sites, in all three cases, very weak fiber labeling was present in layers 1–2 in both DLO1 and DLO2, although densest in the dorsal DLO1 field. No fibers were seen in any other orbitofrontal field. We did not observe labeling in the medial prefrontal cortex in any of these cases.

The above-described results can be summarized as follows: parietal GI projected preferentially to superficial layers of the dorsolateral orbitofrontal cortex, with a preference for area DLO1 over DLO2. A light projection was noted to reach area LO. Projections to medial prefrontal areas were not observed in our material. This projection pattern is thus similar to that seen in case of parietal DI. The two cases with dense projections to the DLO field were the only cases with substantial involvement of layer 2 in the injection sites which suggests that the projections originate from this layer.

### AIP

In three cases, the tracer deposit was centered in AIP. In case 14704B, the injection was restricted to AIP, except for a few single labeled cells ventrally, potentially in the piriform cortex. In the two other cases, the injections showed either a very small (12949B) or a substantial (18075B) involvement of the parietal DI. The superficial layers were densely involved in all three injection sites, and in case 18075B layer 5 was involved as well. In all three cases, the same overall pattern of labeling was observed. In the infra- and prelimbic cortices, only very weak labeling was present, whereas a very dense plexus was labeled in the dorsal peduncular cortex. In the orbital fields, moderate or dense labeling was present in the dorsolateral field with substantially weaker labeling in the lateral field.

**Figure 9:**
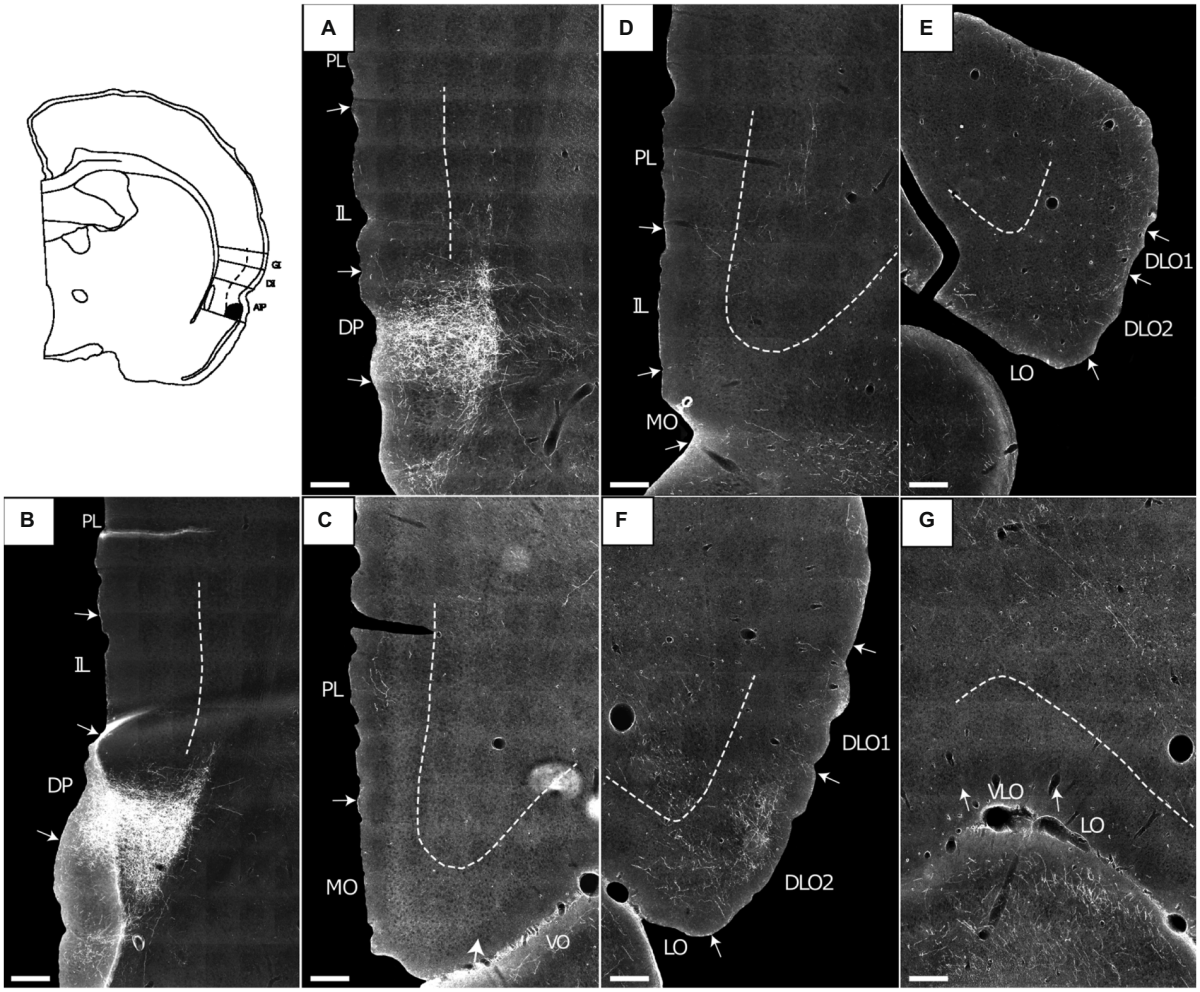
Images showing the fiber projections from a BDA injection restricted to the superficial layers of the agranular parietal insular cortex (case 14704B). A schematic depicting the injection site is shown in the upper left corner. **(A,B)** A very dense and restricted fiber plexus is present in the dorsal peduncular cortex (DP), here shown in two nearby sections. The anterograde labeled fibers extend only very sparsely into the infralimbic cortex (IL). **(C,D)** Only occasional scattered fiber labeling is present at more rostral portions of the prelimbic (PL) and infralimbic (IL) cortices, as well as the medial orbital field. **(E,F)** Fibers innervate superficial layers of the dorsolateral orbital field (DLO), particular DLO2, while substantially sparser labeling is present in the lateral orbital field (LO). **(G)** The ventral (VO) and ventrolateral (VLO) orbital fields are virtually devoid of fiber label. Dashed lines indicate the border between layers 3 and 5. Scale bars equal 250 μm.

In the representative case 14704B, with the tracer deposit being restricted to the superficial layers of AIP, we observed strong labeling at caudal levels in the superficial cell layer of the dorsal peduncular cortex. This labeled plexus diminished abruptly at the border to the infralimbic cortex, which only had scattered fibers except for a rather weakly labeled and very narrow plexus in the deep layers (Figures 9A,B). At more rostral levels, scattered labeling was observed primarily in the deep layers of the prelimbic cortex, although at even more rostral levels, additional fibers were labeled in layers 1–2 (Figures 9C,D). Only very few randomly dispersed fibers were labeled in the infralimbic cortex at these rostrocaudal levels. In the anterior cingulate cortex, only a few scattered fibers were labeled, primarily in layer 1. In the dorsolateral orbital field, a moderately labeled plexus was present in DLO2 with substantially weaker labeling dorsally in DLO1. Labeling was mainly present in layer 1, with some in layer 2, and only weak terminal labeling in layer 5 (Figures 9E,F). In the lateral orbital field, only scattered fibers were present in layer 1, although at most caudal levels the labeling was slightly denser and also included the deeper layers (Figures 9E,F). Virtually no labeling was observed in other orbital fields (Figures 9C,G).

**Figure 10:**
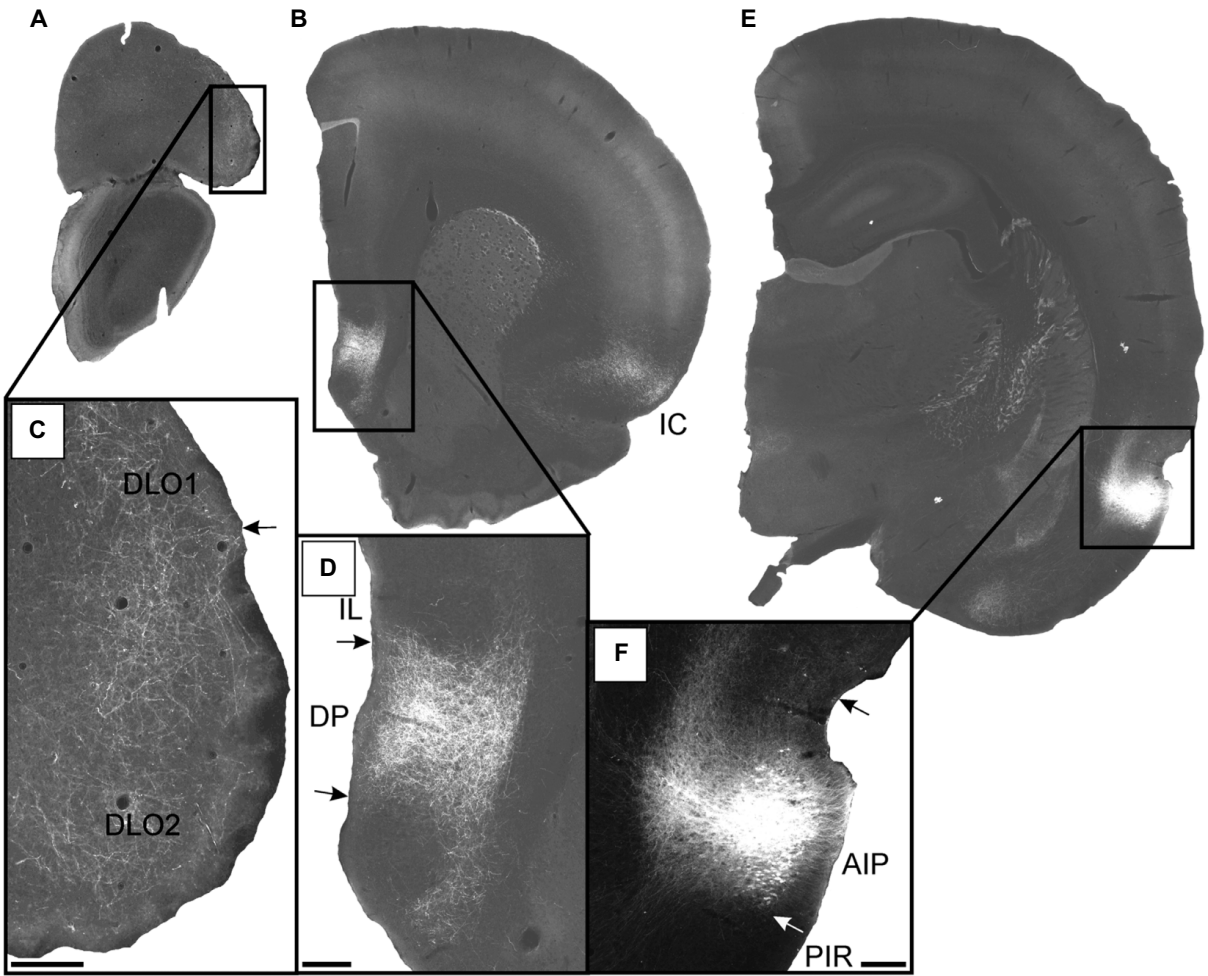
Images visualizing the fiber projections from the agranular field of the parietal insular domain (case 12949B). **(A,B)** Low-resolution images of frontal sections with boxes indicating the areas of higher-resolution images shown in **C** and **D**. **(C)** Dense fiber labeling is present in the dorsolateral orbital fields DLO1 and DLO2. **(D)** A densely labeled fiber plexus is present in the dorsal peduncular cortex. **(E)** Low-resolution image of section with box indicating the area of higher-resolution image shown in **F**. **(F)** The center of the BDA injection in the agranular parietal insular cortex. A smaller portion of the tracer deposit extends into the dysgranular parietal insular cortex. AIP, parietal agranular insular field; DLO1, dorsolateral orbital field 1; DLO2, dorsolateral orbital field 2; DP, dorsal peduncular cortex; IC, insular cortex; IL, infralimbic cortex; PIR, piriform cortex. Scale bars equal 200 μm.

In the remaining two cases, the labeling pattern was comparable with some minor differences, primarily in terms of laminar fiber distribution (Figure 10). In DLO, we observed additional very weak labeling of layer 3 and in LO layer 2 showed labeling. In one of these cases (12949B), the ventral portion of DLO1 was also densely innervated (Figures 10A,C). Similar to the representative case 14704B, dense labeling was present in the dorsal peduncular cortex (Figures 10B,D) except that the scattered rostral labeling, seen in the prelimbic cortex, also involved the infralimbic cortex in these cases. In the anterior cingulate cortex, virtually no fibers were present in any of the AIP injected cases.

These results indicate that AIP apparently projected preferentially to the dorsal peduncular cortex and the dorsolateral orbitofrontal field DLO2. Neurons in superficial layers of AIP clearly contributed to these projections but our data do not reveal if the projections also originate from deeper layers of the AIP. Projections to other medial prefrontal and orbitofrontal areas are sparse to absent.

## Discussion

Insular cortex projections to the orbitofrontal cortex are dense and originate from all areas investigated, except for the posterior GI field. In contrast, projections to the medial prefrontal cortex originate almost exclusively from the agranular fields and they target predominantly the ventral portion of the medial prefrontal cortex. Although our data do not allow us to conclude the laminar origin of all insular projections, they do suggest that for most insular regions a dense component of these projections originate from layer 2, but deeper layers clearly contribute to the projections too. The data do not allow to infer whether there are specific differences in origin between the various insular regions. In the subsequent sections we will discuss the insular cortex projections in more depths to address the questions/hypothesis formulated in the introduction.

### Projections to the orbitofrontal cortex

**Figure 11:**
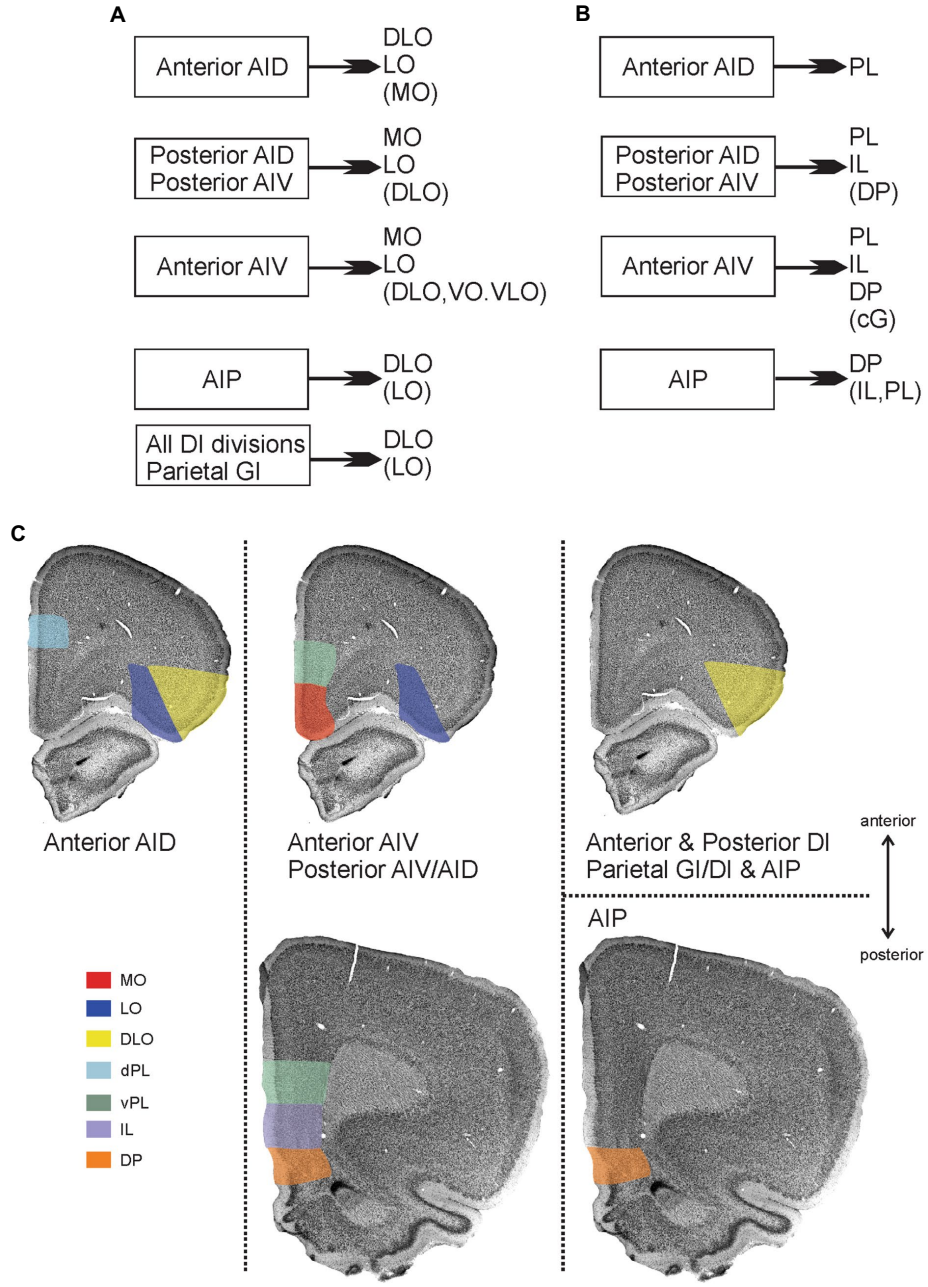
Main patterns of projections from insular cortex to orbitofrontal and medial prefrontal cortex. **(A)** Schematic diagram summarizing projections from distinct insular fields to the orbitofrontal cortex. **(B)** Schematic diagram summarizing projections from distinct insular fields to the medial prefrontal cortex. In both A and B, weaker projections are listed within brackets. **(C)** Visualization of the projections to orbitofrontal and medial prefrontal cortices, including the more detailed dorso-ventral and anterior–posterior organization of projections to the medial prefrontal areas. Termination of insular fiber pathways in the orbitofrontal and medial prefrontal cortex are indicated by a specific color for each orbital field that receives dense projections (shown in **A,B**). The anterior dorsal agranular insular field (anterior AID) targets the dorsolateral (DLO) and lateral (LO) orbital fields as well as the dorsal portion of the prelimbic cortex (dPL), specifically at rostral levels. The anterior ventral agranular field (anterior AIV), in contrast, targets lateral orbital (LO), medial orbital (MO), ventral prelimbic (vPL) anteriorly, and vPL, infralimbic (IL), and dorsal peduncular cortex (DP) at more posterior levels. These same areas are receiving inputs from both posterior agranular fields (posterior AIV/AID) The agranular parietal insular field (AIP) targets more specifically the dorsolateral (DLO) field and the dorsal peduncular cortex (DP). Finally, all the dysgranular portions, as well as the parietal granular field, provide a projection that is restricted to the dorsolateral field (DLO), avoiding mPFC regions.

Our results, summarized in Figure 11, show that insular projections preferentially target the DLO, LO and MO fields of the orbitofrontal cortex with only very weak innervation of the VO and VLO fields. The main projections originating in anterior AID and AIP, as well as in dysgranular and granular fields, preferentially target the lateral portion of the orbitofrontal cortex, whereas projections arising from anterior AIV and both posterior agranular fields project to both lateral and medial orbitofrontal fields. Within this overall division, more subtle differences are apparent. Anterior AID projects densely to both DLO and LO, whereas AIP projects densely to only DLO and only weakly to LO. The latter innervation pattern in the orbitofrontal cortex is also seen in case of projections from granular and dysgranular insular fields. Note, however, that in these cases the density as well as the degree of innervation of LO differs depending on the different insular subfields of origin. In contrast, projections originating in the anterior AIV and both posterior agranular fields (AIV and AID) innervate DLO only weakly, and specifically target MO and LO. Finally, the weak innervation of the orbitofrontal fields VO and VLO originates almost exclusively in anterior AIV.

Additionally, the projections to DLO show an interesting pattern. All the agranular fields as well as the anterior and posterior DI target specifically the ventral DLO2 field. Projections from the parietal insular domain display a marked topography, such that a dorsoventral axis of origin in the parietal insular domain relates to a dorsoventral axis of innervation of DLO. The ventral field AIP targets the ventral DLO2, the centrally located DI projects to both ventral DLO2 and dorsal DLO1, whereas the dorsal parietal insular domain GI selectively innervates the dorsal field DLO1.

#### Comparison with other studies

Our data agree with previous anterograde and retrograde tracing studies on insular cortex projections in the rat (Reep et al., 1996; Shi and Cassell, 1998b; Jasmin et al., 2004), as well as with an anterograde tract-tracing study on anterior insular efferents in the hamster (Reep and Winans, 1982). However, in none of these studies a complete and systematic analysis of the insular-orbitofrontal projections was provided, whereas in other tracing studies on insular-cortical projections, there is no mention of any orbitofrontal connectivity (Saper, 1982; Yasui et al., 1991).

In contrast to the present findings, Reep et al. (1996) reported dense retrograde labeling in the insular cortex resulting from injections placed in VLO, indicative of a substantial insular innervation of VLO. Since the injection sites in VLO presented in that paper show some involvement of LO, our results thus suggest that it is more likely that the labeled cells in the insular cortex project to LO rather than to VLO. Also, it should be noticed that dense insular projections target areas deep to the VLO, which could be a factor explaining this discrepancy. As described above, our findings additionally document the specific origins of these insular-orbitofrontal projections. With respect to the laminar origin of the projections, according to our study all insular layers contribute to the projections to the orbitofrontal cortex though with a preferential origin from the superficial layers.

#### Functional considerations

An important functional consequence of these findings is that the primary gustatory cortex, essentially positioned in posterior DI, provides a dense projection to the orbitofrontal cortex, specifically to DLO. This contrasts with the lack of a primary gustatory projection to medial prefrontal cortices. The orbitofrontal cortex is an important part of the neural circuit that processes gustatory information in primates, including humans (Rolls, 2000, 2005; Small et al., 2007). Specifically, cells in the caudolateral orbitofrontal cortex of primates respond to gustatory stimuli (Rolls et al., 1990) and this area, sometimes referred to as the secondary gustatory cortex, receives a direct projection from the insular primary gustatory cortex (Baylis et al., 1995; Cavada et al., 2000). In the rat, neural representations of the palatability and reward-related aspects of food are present in the orbitofrontal cortex, which is also involved in aspects of eating behavior and licking (Shipley et al., 1980; Whishaw and Kolb, 1983; Gutierrez et al., 2006; Travers, 2006). Based on the specificity of the primary gustatory cortex projections as shown in this study we, therefore, propose that DLO is an orbitofrontal gustatory area in the rat.

In view of this proposition, it is relevant that taste might function as a primary reinforcer (Rolls, 2000), necessary for association learning in the orbitofrontal cortex (Schoenbaum et al., 2009). The orbitofrontal cortex has consistently been found to be involved in this complex function, which is variably thought to reflect orbitofrontal involvement in response inhibition, fast coding of associative information and/or coding of outcome expectancies (Schoenbaum et al., 2003; Schoenbaum and Roesch, 2005; Furuyashiki and Gallagher, 2007; Schoenbaum et al., 2007, 2009; Sul et al., 2010). The insular cortex processes affective and emotional-related information and if these functions contribute to the aforementioned tasks in the orbitofrontal cortex, the present data suggest that this information is predominantly processed in the LO, DLO and MO fields, not in the VO or VLO fields.

### Projections to the medial prefrontal cortex

The present data lead us to conclude that only agranular insular fields project to the medial prefrontal cortex. None of the dysgranular and granular insular fields contribute in any substantial way to these mPFC projections. We further show that the distribution of insular projections in the medial prefrontal regions displays a striking topological organization (Figure 11). Fibers originating from the parietal AIP field of the insular cortex terminate in a particularly dense plexus in the dorsal peduncular cortex with more scattered fiber label in other mPFC areas. In contrast, anterior AID projections are restricted to the dorsal prelimbic cortex and completely avoid the ventral portion. Projections from the other agranular insular areas show almost an opposite preference for the ventral part of the prelimbic area and include as a target the infralimbic area and adjacent dorsal peduncular cortex. In addition, anterior AIV projects moderately to the anterior cingulate cortex.

The agranular fields of the posterior insular domain densely target the infralimbic and the ventral portion of the prelimbic cortex with a more moderate innervation of the dorsal prelimbic cortex and the dorsal peduncular cortex. Finally, a rostro-caudal organization of insular to medial prefrontal projections is present. Dense projections to the prelimbic cortex always preferentially innervate rostral levels, independently of whether they target ventral or dorsal levels, whereas the infralimbic and dorsal peduncular show a preferred caudal innervation by insular inputs.

### Comparison with previous studies

Taking differences in nomenclatural usage into account, it is apparent that our present results agree with previous findings. Several studies, mostly concerned with overall distribution of insular efferents, reported projections to the medial prefrontal cortex (Reep and Winans, 1982; Saper, 1982; Krushel and Van Der Kooy, 1988; Allen et al., 1991; Yasui et al., 1991; Shi and Cassell, 1998b; Gabbott et al., 2003; Jasmin et al., 2004). However, none of these studies provided a systematic analysis of the insular-medial prefrontal cortex projections. Our data also concur with studies in which medial prefrontal cortex afferents have been analyzed with the use of retrograde tracing techniques (Van Eden et al., 1992; Conde et al., 1995; Hoover and Vertes, 2007). Also, these studies indicate that the insular projections in general originate from all layers, although with a possible preference for the superficial layers in case of infralimbic projections (Hoover and Vertes, 2007).

Irrespective of the overall similarities between all studies, our data bear to some ambiguities that emerge from a systematic comparison between studies. One noticeably example is the conclusion of Shi and Cassell (1998b) that the agranular anterior and the agranular posterior insular cortices selectively project to the prelimbic or the infralimbic cortex, respectively. Other authors concluded that both of these insular divisions project to both the infra- and pre-limbic cortices (Reep and Winans, 1982; Hoover and Vertes, 2007; Zingg et al., 2014). Our data support the latter conclusion. The preferred terminal distribution, in our data, relates to the origin in the AID versus AIV fields and since Shi and Cassell (1998b) did not differentiate between these two, this may be the cause of the discrepancy in the reported data.

Yasui et al. (1991) claimed that a rostral portion of the parietal insular domain projects densely to a ventral portion of the medial prefrontal cortex with only very weak projections originating from more caudal levels. This ventral portion was designated the infralimbic cortex, but clearly correspond to the dorsal peduncular cortex, according to our terminology. This claim contrasts with our findings, but since the caudal origin in the latter paper (see Figure 7; Yasui et al., 1991) is in the DI field, which indeed does not project densely to the medial prefrontal cortex, we conclude that the two data sets are corroboratively indicating that medial prefrontal input originate mainly from agranular insular divisions.

Finally, one of our main findings, that AIP preferentially projects to the dorsal peduncular cortex, is in agreement with retrograde tracing studies showing that the most caudal portion of the insular cortex do not give rise to projections to the infralimbic, prelimbic and anterior cingulate cortices (Krushel and Van Der Kooy, 1988; Conde et al., 1995; but see Hoover and Vertes, 2007). In line with this, in a study in the mouse it was also reported that the AIP field specifically targets the dorsal peduncular cortex (Zingg et al., 2014).

### Functional considerations

The present study shows that the medial prefrontal cortex is targeted by strong projections originating only from the agranular components of the insular cortex. Insular areas that do not contribute to the projections to the medial prefrontal cortex include posterior dysgranular and granular cortices. Functionally the latter insular areas represent primary gustatory and viscerosensory information processing domains.

The dorso-ventral fiber distribution in the mPFC does not specifically follow the infra- and pre-limbic borders. This points to an important aspect of the functional organization of the mPFC, since it indicates that hodological patterns do not adhere to the cytoarchitectural defined partition of mPFC into four areas (Sesack et al., 1989; Hurley et al., 1991; Van Eden et al., 1992; Datiche and Cattarelli, 1996; McDonald et al., 1996; Heidbreder and Groenewegen, 2003; Gabbott et al., 2005; Vertes, 2006). Rather, a functional differentiation between dorsal and ventral portions of the medial prefrontal cortex is more appropriate (Heidbreder and Groenewegen, 2003; Euston et al., 2012). In this respect, if we look at all insular domains and fields combined, insular cortex targets the dorsal portions (dorsal PL and Cg) less than the ventral portions, indicating that insular cortex might have a rather limited influence on temporal patterning of behavioral sequences (Heidbreder and Groenewegen, 2003).

In contrast, ventral prelimbic and infralimbic cortex share dense insular inputs that preferentially arise from the anterior AIV as well as both posterior agranular fields. The shared inputs from the posterior agranular areas likely represent a viscerosensory-visceromotor pathway (Gabbott et al., 2003), with viscerosensory information reaching mPFC via the insular cortex (Cechetto and Saper, 1987; Allen et al., 1991; see also Hurley-Gius and Neafsey, 1986; Hardy and Holmes, 1988; Owens et al., 1999; Fisk and Wyss, 2000).

The dorsal peduncular cortex receives strong projections from the agranular parietal field AIP. Although this projection extends as a weak to moderate projection to the directly adjacent ventral part of the infralimbic cortex, we suggest that insular projections differentiate the dorsal peduncular cortex from the infralimbic cortex. Interestingly, this organizational pattern does not seem to be unique for the insular cortex. The hippocampus and the parahippocampal region, known to contribute dense projections to the medial prefrontal cortex (Jay and Witter, 1991; Van Eden et al., 1992; Conde et al., 1995; Delatour and Witter, 2002; Cenquizca and Swanson, 2007; Hoover and Vertes, 2007), show a similar terminal preference in that specific parts of the entorhinal cortex project densely to the dorsal peduncular cortex, and avoid the infra- and prelimbic cortices (Haberly and Price, 1978; Insausti et al., 1997). Other projections that densely target the dorsal peduncular cortex originate from the anterior piriform cortex, the olfactory bulb and the olfactory tubercle (Datiche and Cattarelli, 1996). Consequently, anatomical data point to the dorsal peduncular cortex as a zone of convergence between parietal insular, olfactory and entorhinal projections. The parietal insular cortex integrates somatosensory and auditory information and has been implicated in auditory fear conditioning (Rosen et al., 1992; Shi and Davis, 1999; Brunzell and Kim, 2001; Kimura et al., 2007). It thus seems likely that this information is integrated with olfactory and memory-related information in the dorsal peduncular cortex.

### Summary

Insular ‘primary’ sensory cortices, encoding gustatory and visceral sensory modalities virtually avoid mPFC. In contrast, these insular projections strongly innervate OFC, specifically targetting DLO. The latter might thus be considered a secondary gustatory cortex. Insular agranular areas target both mPFC and OFC. In mPFC, these projections target the ventral prelimbic and adjacent infralimbic areas, as well as the dorsal peduncular cortex, though the latter receives the strongest inputs from the “parietal” domains of the insular cortex. In OFC, the agranular insular projections densely innervate medial and lateral fields, with only weak to absent innervation of the ventral fields VO/VLO. As taste-responsive and licking-modulated neurons have been reported in the mPFC (e.g., Horst and Laubach, 2013; Jezzini et al., 2013) our data seem to indicate that relevant information most likely reaches the mPFC indirectly via agranular insular fields.

## Data availability statement

The original contributions presented in the study are included in the article/supplementary materials, further inquiries can be directed to the corresponding author.

## Ethics statement

The animal study was reviewed and approved by the Norwegian Food and Safety Authority, in accordance with the Norwegian Experiments on Animals Act and the European Communities Council Directive.

## Author contributions

MM carried out all experimental work and analyses and wrote the draft of the manuscript. The experiments were designed by MW and MM. MW and JA obtained funding. All authors contributed to discussion and interpretation of the results and to the final version of the manuscript.

## Funding

This study was supported by The Kavli Foundation and the Norwegian Research Council: The Centre of Excellence scheme—Centre for Neural Computation#223262 and research grant #227769.

## Conflict of interest

The authors declare that the research was conducted in the absence of any commercial or financial relationships that could be construed as a potential conflict of interest.

## Publisher’s note

All claims expressed in this article are solely those of the authors and do not necessarily represent those of their affiliated organizations, or those of the publisher, the editors and the reviewers. Any product that may be evaluated in this article, or claim that may be made by its manufacturer, is not guaranteed or endorsed by the publisher.
